# Systematic Review and Meta-analysis on the Incidence, Prevalence and Determinants of Discomfort in Inflammatory Bowel Disease

**DOI:** 10.1093/jcag/gwab043

**Published:** 2021-11-14

**Authors:** Oana-Irina Nistor, Christina Godfrey, Amanda Ross-White, Rosemary Wilson

**Affiliations:** Faculty of Health Sciences, School of Nursing, Queen’s University, Kingston, Ontario, Canada; Faculty of Health Sciences, School of Nursing, Queen’s University, Kingston, Ontario, Canada; Queen’s Collaboration for Health Care Quality: A JBI Centre of Excellence, Queen’s University, Kingston, Ontario, Canada; Faculty of Health Sciences, School of Nursing, Queen’s University, Kingston, Ontario, Canada; Queen’s University Library, Kingston, Ontario, Canada; Faculty of Health Sciences, School of Nursing, Queen’s University, Kingston, Ontario, Canada; Queen’s Collaboration for Health Care Quality: A JBI Centre of Excellence, Queen’s University, Kingston, Ontario, Canada

**Keywords:** *Determinants*, *Discomfort*, *Inflammatory bowel disease*, *Incidence*, *Prevalence*, *Systematic review*

## Abstract

**Background:**

The symptom burden in inflammatory bowel disease (IBD) has a significant negative impact on the health-related quality of life (HRQOL). Patients with IBD report physical, psychological and social discomfort even during remission.

**Aim:**

To synthesize the best available evidence to determine the worldwide incidence, prevalence and determinants of discomfort in adults with inflammatory bowel disease (IBD).

**Methods:**

Following PRISMA recommendations, we searched the Medline, CINAHL, PsycInfo, Embase, Cochrane, Campbell and JBI Evidence Synthesis databases for studies on either incidence or prevalence of discomfort in English until January 2021. Data were extracted using the Joanna Briggs Institute’s standardized extraction tools. Data that directly reported or could be used to calculate the incidence and prevalence of discomfort were extracted. Ten studies were eligible for inclusion in this review. Overall, the methodological quality of the included studies was considered moderate. Data measuring the incidence of discomfort in 6 out of 10 identified studies using the same measurement tool (EQ-5D) were pooled in a meta-analysis. Additional results have been presented in a narrative form, including tables.

**Results:**

There is no standardized definition or tool utilized to describe or measure discomfort in IBD. Synthesized findings demonstrate that discomfort is prevalent among adults living with IBD. Determinants of discomfort included health literacy, disease activity, hospitalization/surgery, age and gender, delayed diagnosis, local practice standards and quality of IBD care.

**Conclusions:**

More research is needed to identify the impact of discomfort on health-related outcomes for people with IBD and consequently appraise discomfort interventions for their efficacy.

## INTRODUCTION

Inflammatory bowel disease (IBD) has overwhelming consequences on the health-related quality of life and health care utilization (1). It has been estimated that IBD affects five million people worldwide (2), with a prevalence of 0.5% in North America and a rising incidence posing concerns for an emerging epidemic (3) with unsustainable long-term care (4,5). The complex pathogenesis of IBD leads to a life-long, unpredictable, relapsing and remitting illness course (6,7). The symptom clusters in IBD (pain, discomfort, anxiety and depression) are comparable to those in cancers and have been associated with a decreased health-related quality of life (HRQOL) (1). Thus, there is a critical need to understand the broader impact of the multidimensional nature of IBD discomfort.

There is no published systematic review on the incidence, prevalence, and determinants of discomfort in IBD. An increased understanding of the incidence and prevalence of discomfort would be invaluable for IBD care providers and policymakers to develop effective strategies to manage IBD, provide targeted support for individuals, and address healthcare systems’ implications. Therefore, it is necessary to synthesize the findings of studies conducted on this area to appraise the strengths and limitations of such studies and identify evidence on the prevalence and incidence of discomfort among adults with IBD.

### Objective

The aim of this review was to synthesize the best available evidence to determine the worldwide incidence, prevalence, and determinants of discomfort in adults with IBD. Therefore, the following research questions are addressed: (1) What is the global incidence of discomfort in adults with IBD? (2) What is the global prevalence of discomfort in adults with IBD? and (3) What are the determinants of discomfort in IBD?

### Inclusion and Exclusion Criteria

#### Participants

This review considered studies conducted worldwide involving adults (18 years and older) with IBD reporting discomfort. Studies examining the experience of discomfort in children with IBD have been excluded.

#### Concept

The concept of discomfort has been recognized as a component of illness and suffering, and its conceptualization is essential for measurement (8). The definition of discomfort used to select studies was that of ‘a negative physical and/or emotional state, causing unpleasant feelings or sensations’ (9). Although a familiar concept in clinical practice, research studies have misused discomfort as a surrogate for pain (9). However, while pain can lead to discomfort, not all discomfort is a consequence of pain (9).

The deleterious impact of discomfort on patient outcomes has been previously documented. For example, anal pain or discomfort has been reported as the most critical factor in Crohn’s disease (10). Abdominal discomfort has been described in the context of disease severity, frequent surgical interventions, and hospitalizations (11), abdominal cramping and diarrhea (12). Other sources of discomfort include perianal disease (13), abdominal lump (14), changes in bowel function (15), abdominal distention and flatulence (16), decreased appetite, nausea, vomiting, abdominal tenderness, difficulty passing gas, and sleep disturbance (14,17), food triggers and restrictions (18,19), and dietary concerns (20). Additional sources of discomfort include emotional, relational, familial and employment-related challenges (21), restless leg syndrome (17), worrying about discomfort even in the absence of actual symptoms (22), talking about the negative aspects of the disease (23), lifestyle limitations, social interactions (15) and lack of accommodation by others (24).

In women with IBD, vulvar and vaginal discomfort consisting of pruritus, burning and irritation (25) and sexual dysfunction (26) have been associated with decreased functional status and lower scores on Patient-Reported Outcomes Measurements Information System (PROMIS) measures of emotional and mental health (25). Furthermore, physical and psychological discomfort persist in IBD even in remission (27,28). Therefore, it is imperative to concede, evaluate, and prevent the sources of discomfort to provide patient-centred IBD care (29).

This review primarily considered studies that assessed and reported on the incidence and prevalence of discomfort in IBD. The symptom of discomfort was captured as reported within the studies. Those with concept confusion, where discomfort was not defined or was used to describe pain in the context of endoscopic or imaging evaluations, have been excluded.

#### Context

This review considered worldwide studies conducted in adult, inpatient, or outpatient IBD care settings.

#### Types of Studies

Studies with an observational design, including prospective and retrospective cohort studies, case–control and cross-sectional studies and surveys published worldwide in English involving adults with IBD at any stage in their disease continuum, were appraised in this review. Single case studies have been excluded. In addition, although cohort studies with a prospective or longitudinal design are considered the best in establishing a condition’s incidence or natural history, any studies providing prevalence and incidence information have been examined in this review, regardless of design (30).

## METHODS

**Figure 1.**
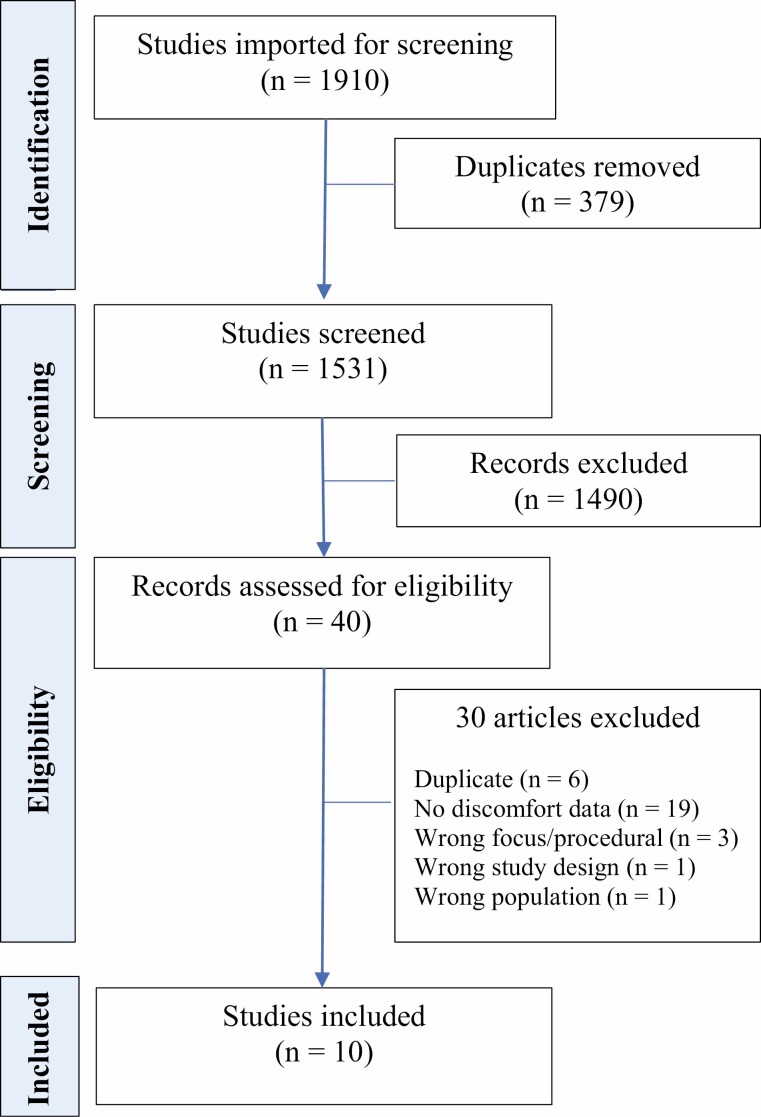
PRISMA flow diagram of study selection and inclusion process (31).

A systematic review of studies was conducted following the Preferred Reporting Items for Systematic reviews and MetaAnalysis (PRISMA) (31) (Figure 1), using the JBI methodology for systematic reviews of prevalence and incidence (30). The protocol of this review was registered in PROSPERO (CRD42021226061) (32) on February 25, 2021.

### Search Strategy

The essential information sources in this systematic review included Medline (Ovid), CINAHL (Ebsco), PsycInfo (Ovid), Embase (Ovid), Cochrane (Wiley), Campbell and JBI Evidence Synthesis (see Supplementary Appendices I and II). The search strategy was designed and conducted in collaboration with an experienced scientific librarian and aimed to identify the published and unpublished studies written in English. The included search terms were ‘discomfort’, ‘inflammatory bowel disease’, ‘Crohn’s’ and ‘colitis’.. The search for unpublished studies and grey literature (33) included trial registers and ProQuest Dissertations and Theses. The electronic searches were enhanced with hand-searching of reference sections from studies retrieved via databases. Databases were searched from their inception to January 2021. Next, the reference lists of all reports and articles selected for critical appraisal have been screened for additional studies. Finally, an author search has been conducted on the authors’ names known to have researched the review objective.

### Study Selection

All the identified citations have been collated and uploaded into Covidence systematic review software (Veritas Health Innovation, Melbourne, Australia) (34) and duplicates removed. Following a pilot test, the full text of selected citations has been assessed in detail against the inclusion criteria by two independent reviewers (O.I.N., R.W.), with arbitration about final inclusion from a third reviewer (C.G.) where required.

### Data Extraction and Analysis

Potentially relevant studies have been retrieved in full, and their citation details were imported into the Unified Management, Assessment and Review of Information (JBI SUMARI; JBI, Adelaide, Australia) (35). A priori protocol guided this review, with the intent to pool the included studies, where possible, in a statistical meta-analysis using JBI SUMARI. The selected studies were then assessed for methodological quality by two independent reviewers using the standardized critical appraisal instrument for prevalence studies (Joanna Briggs Institute Critical Appraisal Checklist for Studies Reporting Prevalence Data) (30) and the critical appraisal checklist for cohort studies (36). Due to the variability across studies, attributed to methodological, clinical, geographical and statistical differences, the critical appraisal results are presented in Tables 1 and 2 with an accompanying narrative.

**Table 1.** Critical appraisal of included prevalence studies (30)

Citation	Q1	Q2	Q3	Q4	Q5	Q6	Q7	Q8	Q9	%
Armuzzi et al. 2019.	Y	Y	Y	Y	Y	Y	Y	Y	Y	100
Carels et al. 2019.	Y	Y	Y	Y	Y	Y	Y	Y	Y	88.88
Ding et al. 2019.	Y	Y	Y	Y	Y	Y	Y	Y	U	88.88
Lee et al. 2017.	Y	Y	Y	Y	Y	Y	Y	Y	Y	100
Obando et al. 2019.	Y	Y	Y	Y	Y	Y	Y	Y	U	88.88
Ona et al. 2020.	Y	Y	Y	Y	Y	Y	Y	Y	Y	100
Petryszyn et al. 2015.	Y	Y	Y	N	U	U	U	U	U	33.33
Piercy et al. 2015.	Y	Y	Y	Y	Y	Y	Y	Y	U	100
Yan et al. 2020.	Y	Y	Y	Y	Y	Y	Y	Y	Y	100
Total (%) Yes	100	100	100	88.88	88.88	88.88	88.88	88.88	55.55	

N, no; U, unclear; Y, yes.

Q1: Was the sample frame appropriate to address the target population?

Q2: Were study participants sampled in an appropriate way?

Q3: Was the sample size adequate?

Q4: Were the study subjects and the setting described in detail?

Q5: Was the data analysis conducted with sufficient coverage of the identified sample? Q6: Were valid methods used for the identification of the condition?

Q7: Was the condition measured in a standard, reliable way for all participants?

Q8: Was there appropriate statistical analysis?

Q9: Was the response rate adequate, and if not, was the low response rate managed appropriately?

**Table 2.** Critical appraisal of the included cohort study (36)

Citation	Q1	Q2	Q3	Q4	Q5	Q6	Q7	Q8	Q9	Q10	Q11	%
Shah et al. 2018.	Y	Y	Y	N	N	U	Y	Y	U	N	U	45.45
%	100	100	100	0	0	0	100	100	0	0	0	

N, no; U, unclear; Y, yes.

Q1: Were the two groups similar and recruited from the same population?

Q2: Were the exposures measured similarly to assign people to both exposed and unexposed groups?

Q3: Was the exposure measured in a valid and reliable way?

Q4: Were confounding factors identified?

Q5: Were strategies to deal with confounding factors stated?

Q6: Were the groups/participants free of the outcome at the start of the study (or at the moment of exposure)?

Q7: Were the outcomes measured in a valid and reliable way?

Q8: Was the follow up time reported and sufficient to be long enough for outcomes to occur?

Q9: Was follow-up complete, and if not, were the reasons to loss to follow-up described and explored?

Q10: Were strategies to address incomplete follow-up utilized?

Q11: Was appropriate statistical analysis used?

## RESULTS

After the databases were searched and duplicates were removed from retrieved records, 1531 titles and abstracts were screened. Articles that did not fit the inclusion criteria were excluded resulting in 40 citations identified as appropriate for detailed, full-text assessment. Thirty studies were excluded after full-text evaluation. Data were only included once for studies reporting on the same participants (*n* = 4). The remaining 10 articles were selected for critical appraisal.

### Characteristics of Included Studies

The final 10 studies included in this systematic review consist of two prospective observational cohort studies (29,37), two retrospective chart review and patient self-completion questionnaires (38,39), one retrospective observational study (40), a cross-sectional study design (online survey) (41,42), one large, multinational, cross-sectional survey (43), a cohort study (44) and a multicenter, observational study (45). In addition, the authors have been contacted where data were missing to seek further clarifications (*n* = 6), with one response (41) providing more results. The characteristics of the ten included studies are shown in Table 4. The data extracted included specific details about the participants, condition, other characteristics and outcome information, including the proportion of people reported with either current or period or lifetime prevalence of discomfort in IBD or outcome data. Only the baseline data were extracted in cohort study designs that measured the prevalence of discomfort with multiple data points.

A total of 9384 patients diagnosed with IBD (5067 Crohn’s disease [CD], 4317 ulcerative colitis [UC]/indeterminate colitis) from 10 studies were included in this review. The participants in the included studies were from Australia (44), Belgium (37), China (45), France (43), Germany (43), Italy (29,43), Poland (42), South Korea (40), Spain (43) and the USA (38,39,41,43). Sample sizes ranged from 52 (37) to 2093 (44) participants with IBD, and the proportion of female participants ranged from 19 (37) to 1250 (41). The age of participants ranged from 18 to 84 years. Two studies enrolled participants from outpatient settings (29,45), IBD patient registry (37), online platforms (41,42), multinational participants (43) and multicenter participants (45). The observational period ranged from 2 (43) to 180 months (40). One study did not specify the observational period (42). Three studies collected data retrospectively (38–40), three collected data prospectively (29,37,45), three were cross-sectional studies (41–43), and one was a cohort study (44). Participants in remission ranged between 19% (38) and 55.44% (43).

### Methodological Quality

All the 10 included studies underwent critical appraisal, data extraction and synthesis to capture valuable insights regardless of their methodological quality, to gain a richer understanding of discomfort. As a result, no study was excluded based on the quality assessment. However, the nine included prevalence studies scored between 8 to 9 out of 9 on the Critical Appraisal Checklist for Studies Reporting Prevalence Data (30) (Table 3). One cohort study (44) scored 5 out of 11 on the critical appraisal checklist for cohort studies (36). Five out of ten studies answered ‘Yes’ for each critical appraisal question, meaning that the risk of bias across included studies was moderate. The reviewers ensured that the participants in the included studies were representative of the target population and were adequately recruited. Most of the included studies provided adequate details about their participants and study settings. However, one study did not specify the observation period (42).

**Table 3.** Data extraction instrument (46)

The unmodified JBI Data extraction form for prevalence studies
Citation Details
Authors:
Title:
Journal:
Year:
Issue:
Volume:
Pages:
Generic Study details
Study design:
Country:
Setting/Context:
Year/ timeframe for data collection:
Participant Characteristics (study inclusion/exclusion information):
Condition and measurement method:
Description of main results (n/N):

### Discomfort Measurement Instruments

Six (29,37–39,42,43) out of the ten included studies used the standardized, Component 1 of the EuroQoL 5 Dimensions (EQ-5D) to measure discomfort. The EQ-5D is a non-disease-specific instrument that evaluates HRQoL while capturing the patients’ experiences with pain/discomfort, rated from 1 = no problems to 3 = severe/extreme problems (29). While the psychometric properties of EQ-5D have been established, EQ-5D does not differentiate further between pain and discomfort and lacks the required sensitivity to differentiate further at the subgroups analysis (42).

One study reported the number of patients with perianal discomfort as a chief complaint without specifying a measuring tool (40). Another study reported the number of women with IBD experiencing vulvar and vaginal discomfort based on the patients’ response to a vulvovaginal symptom survey which included three separate questions about feeling itching, burning or irritation in the past month (41). However, no specific phenotype of vulvovaginal discomfort was found to be dominant (41). A single study used the validated Structured Assessment of Gastrointestinal Symptoms (SAGIS) to assess the severity of pain/discomfort at defecation on a 5-point scale (44). Finally, the last included study described using the ‘Standard Set of Patient-centered Outcomes for Inflammatory Bowel Disease – an International, Cross-disciplinary Consensus’, a prestandardized set of patient-centred outcome measures for IBD developed by an international working group (45).

### Prevalence of Discomfort

The prevalence of discomfort was synthesized for each included study. The prevalence was expressed as the proportion/percentage of study participants with discomfort as determined by the measuring tool. Data on the number of patients with IBD reporting discomfort ranged from 7.5% of women with IBD reporting vaginal discomfort while in remission (41) to 88.7% of patients with moderate to severe Crohn’s disease (39) (Table 4).

**Table 4.** Summary of included studies

Study/ Country/ Time period/ Design	Aim of study	Characteristics/ setting	Definition and Data Sources	Prevalence of Discomfort	Determinants of Discomfort
Armuzzi et al., 2019, Italy, Feb2012–Nov2013, a prospective observational cohort study	To assess disease evolution and its relationship with work ability-efficiency, disease activity, disease-related worries, treatment satisfaction and compliance.	*N* = 552 CD, Age in years, median (range) (41 (18–84)). Female gender n (%) 271 (49). Years from CD diagnosis, median (range) (6 (0–49)). Harvey-Bradshaw Index, median score (range) (9 (8–39)). Routine outpatient visits.	EuroQoL 5 Dimensions (EQ-5D) component 1: pain/discomfort	CD only: -Baseline 87% - M3 67% - M6 60% - M12 58%	-
Carels et al., 2019, Belgium, May 1st, 2008, to April 30th, 2010, with subsequent five years, prospective and observational study	To determine health literacy (HL), quality of life (QoL) and clinical outcomes in young adults from the BELgian CROhn’s disease registry (BELCRO) in comparison to type 1 diabetes mellitus (DM) as a control.	*N* = 52 CD. Median age (IQR) years 25.0 (23.8–27.0). Female gender (%) 19 (36). Median CDAI (IQR) 76.5 (20.2–135.9). Remission (CDAI <150): 39 CD patients. Active disease (median CDAI 251.8 [205.9–307.0], maximum value of 415.6): 2 CD patients.BELgian CROhn’s disease registry (BELCRO).	EuroQoL 5 Dimensions (EQ-5D) pain/discomfort	CD only: - 50% *with* hospitalization/ surgery-54%*without*hospitalization/ surgery 49%	- Health Literacy - Disease activity - Hospital/surgery
Ding et al., 2019, US, 2015 and 2017, a real-world survey of gastroenterologist-completed retrospective chart review and patient self-completion questionnaires	To establish the association between Patient-Reported Outcomes (PROs) and disease severity measured by partial Mayo score (PMS) using real-world data.	*N* = 1389 UC. Remission (410): mean age of 44.8 years, 48.8%, female, 7.0 mean years since diagnosis. Mild (646): mean age 42.6 years, 52.2% female, 4.5 mean years since diagnosis. Mod/sev (333): mean age of 39.4 years, 45.9% female, 3.0 mean years since diagnosis. Retrospective chart review and patient self-completion questionnaires.	EuroQoL 5 Dimensions (EQ-5D) pain/discomfort	UC only: -Remission 19%, - Mild 52.7% - Mod/sev 69.9%	Disease activity
Lee et al. 2017, South Korea, from January 2000 to December 2015, retrospective observational design.	To investigate the factors affecting diagnostic delay and outcomes of diagnostic delay in IBD.	*Crohn’s patients*: *N* = 165, delayed 41, non-delayed 124, female 39 (23.6%), perianal disease: total 31(18.8%), delayed (12.2%), non-delayed 26 (21%). *UC patients: N* = 130, delayed 32, non-delayed 98; female 59 (45.4). Mayo score at diagnosis was 5.60 ± 1.98. Remission 2 (1.5%), Mild 53 (40.8%), Moderate 74 (56.9%). Retrospective analysis of existing hospital medical records.	Perianal discomfort(chief complaint)	CD: - Total 15.2% - Delayed diagnosis 24.4% - Non-delayed 12.1% UC:**-**	-Age - Delayed diagnosis - Local practice standards
Obando et al., 2019, US, 2015 and 2017, a real-world survey of gastroenterologist-completed retrospective chart review and patient self-completion questionnaires	To establish the association between PROs and the Crohn’s Disease Activity Index (CDAI) using real-world data.	*N* = 468 CD. Remission (291), Mild (105), Mod/sev) 72. Mean duration of 7.5 (remission), 6.9 (mild) and 5.5 (mod/sev) years since diagnosis. Retrospective chart review and questionnaires, Adelphi Disease Specific Programme.	EuroQoL 5 Dimensions (EQ-5D) pain/ discomfort	CD only: - Remission 29.4% - Mild 72.1% -Mod/sev 88.7%	
Ona et al., 2020, US, September 2015 to March 2016, cross-sectional design (online survey)	To define the prevalence of vulvovaginal symptoms and association with IBD activity in a large cohort of women.	*N* = 1250 women with IBD, > 18 years old. *Symptomatic:*n=512, CD 334 (65), UC 178 (35), Age, y 41 ± 14, Ostomy 13 (3), Steroids 66 (13),Immunosuppressants 117 (23), Biologic agents 242 (47). *Asymptomatic: n* = 738, CD 471 (64), UC/indeterminate colitis 267 (36), Age, y 40 ± 14, Ostomy 6 (1), Steroids 65 (9), Immunosuppressants 117 (23), Biologic agents 301 (41). CFA Partners, ‘ ‘Crohn’s and Colitis Foundation online cohort.	Vulvar and vaginal discomfort (vulvovaginal symptom survey)	*Active IBD* - Vulvar discomfort (20.4%) - Vaginal discomfort (9.3%) *Remission:* - Vulvar discomfort (17.1%) - Vaginal discomfort (7.5%)	Disease activity
Petryszyn et al, 2015, Poland, online survey	The aim of this study was to measure HRQoL with the use of the EQ-5D and to compare it with the self-evaluation of health state in patients with inflammatory bowel disease (IBD).	*N* = 169 IBD patients. Female 93 (55%), male 76 (45%). Mean age of respondents was 29.9 ± 8.98 years (range: 18 to 61). UC 73, CD 84.Time since diagnosis: within 1 year (40 patients), 1–9 years (91 patients), at least 10 years since diagnosis (38).Surgery43 patients	EuroQoL 5 Dimensions (EQ-5D) pain/discomfort	72.78% (All IBD)	-
Piercy et al., 2015, France, Germany, Italy, Spain, and the US, July to September 2012, large, multinational, cross-sectional survey	To assess whether patients with Crohn’s disease are more affected by their disease than patients with UC.	*N* = 2065 pts (981 UC, 1084 CD). Mean age 39.4 years (UC) vs. 39.6 year (CD). Female gender: 50.8% UC vs. 50.5% CD. Moderate/severe UC(87.4%/12.6%) vs. CD(85.4%/14.6%). Adelphi Disease Specific Programme.	EuroQoL 5 Dimensions (EQ-5D) pain/discomfort	UC 51.88% CD 55.44%	-
Shah et al., 2018, Australia, 12-month period, Cohort study.	To determine and compare the proportion of patients with severe or very severe impairment of QoL due to specific GI symptoms in patients with selected highly prevalent GI diseases in the outpatient setting of a tertiary hospital.	Population: 10,000 consecutive occasions of service (OOS). 1,157 CD, 936 UC, 999 FGIDs, 173 cured HCV, 150 GI cancers, 136 CeD and 173 cured HCV. Tertiary teaching hospital.	Pain/ discomfort at defaecation (Structured Assessment of Gastrointestinal Symptoms (SAGIS))	CD 12.2% UC 11.5%	-
Yan et al., 2020, China, March 2016 to November 2017, multicenter, observational study	To determine patient perspectives on the effect of IBD and features of patients with lower satisfaction level and compare patient and physician perception of IBD-related Quality of Care (QoC).	*N* = 891:CD 522 (58.6%), UC 363 (40.1%), IBDU 6 (0.7%). Female gender 362 (41%), CD 201 (38.5%), UC 164 (45.2%). Age, years (range): 37 (18–69) CD, 44 (18–75) UC; Disease duration since diagnosis, years (range): CD 4.1 (1–21), UC 4.5 (1–25). Abdominal surgery: CD 99 (19%), UC 92 (25.3%). In person and online.	Pain or discomfort (patient -centered outcome measures for IBD)	CD 42.7% UC 48.5%	Quality of IBD care

CDAI, Crohn’s Disease Activity Index; CD, Crohn’s disease; CeD, celiac disease; FGID, functional gastrointestinal diseases; HCV, hepatitis C infection; IBD, inflammatory bowel disease; IBDU, IBD unclassified; Mod/sev, moderate to severe disease; UC, ulcerative colitis.

#### Meta-analysis

**Figure 2.**
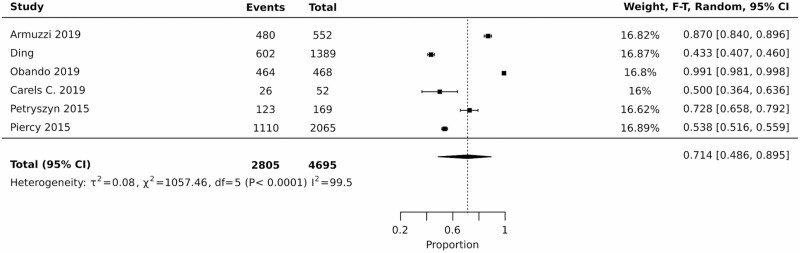
Meta-analysis (forest plot showing point estimates with 95% CI for prevalence of discomfort in IBD) according to the EuroQoL 5 Dimensions (EQ-5D) pain/discomfort.

Since all the included studies used different measurement tools for discomfort, pooling all the studies was not suitable. However, the six studies which measured discomfort using the EuroQoL 5 Dimensions (EQ-5D) component 1 were pooled separately using a random-effects model in JBI SUMARI. The prevalence of discomfort among participants living with IBD was generally high. In the six studies using the EuroQoL 5 Dimensions (EQ-5D) component 1, the prevalence of discomfort ranged from 43.3% to 99.1%. The pooled prevalence was 71.4%, with a 95% CI of 48.6% to 89.5% (Figure 2).

Heterogeneity was determined using the I^2^ statistic and was very high (>99.5%). The number of included studies in the pooled analysis limited the probe of this level of heterogeneity. The observed heterogeneity could be attributed to the definitions for discomfort and its components, the various contexts in which discomfort was measured, and IBD practice standards across several countries, which may have impacted participants’ observed levels of discomfort. The number of studies and data identified in this review prevented us from reporting discomfort rates for any specific subgroups.

### Incidence of Discomfort

Nine out of 10 included studies offered data that permitted the calculation of discomfort rates per person-year, using the sample size, the number of participants reporting discomfort and observation period in months. The discomfort rates in participants with CD ranged from 0.01 per person-year (40) to 3.32 per person-year (43) (1084 participants observed with pain/discomfort). The discomfort rates in participants with UC ranged from 0.19 (38) discomfort per person-year (410 participants observed with pain/discomfort) to 3.11 (43) discomfort per person-year (981 participants observed) (Table 5).

**Table 5.** Discomfort rates of included studies

Study	Type of discomfort (Tool)	Obs. Period months	Sample (*n*)	Sample by IBD Type (*n*)	Discomfort (*n*)	Discomfort rates per person-year*
Armuzzi et al. 2019.	Pain/discomfort (EQ-5D)	22	552	Baseline: CD (535–538)	Baseline (465–468)	0.47
				M3: CD (480–482)	M3 (322–323)	0.33
				M6: CD (457–459)	M6 (274–275)	0.33
				M12: CD (459–462)	M12(266–268)	0.32
Carels et al. 2019.	Pain/discomfort (EQ-5D))	24	52	CD (52)	26	0.25
				CD with hospital. / Surgery (13)	CD with hosp/ surgery (7)	0.26
				CD without hosp/surgery (39)	CD without hosp/surgery (19)	0.24
Ding et al. 2019.	Pain/discomfort (EQ-5D)	24	1389	UC (1389)	602	0.22
				Remission (410)	Remission (153)	0.19
				Mild (646)	Mild (296)	0.23
				Mod/sev: 333	Mod/sev (153)	0.23
Lee et al. 2017.	Perianal discomfort	180	295	CD (165)	25	0.01
				Delayed dx CD (41)	Delayed dx (10)	0.02
				Non-delayed CD (124)	Non-delayed (15)	0.01
				UC (130)		-
Obando et al. 2019.	Pain/discomfort (EQ-5D)	24	468	CD (468)	Remission (289)	0.30
					Mild (104)	0.11
					Mod/sev (71)	0.08
Ona et al. 2020.	Vulvar and vaginal discomfort	7	1250	Active IBD (515)	Vulvar discomfort (105) Vaginal discomfort (88)	0.20 0.30
				Remission (616)	Vulvar discomfort (57) Vaginal discomfort (46)	0.16 0.12
Petryszyn et al, 2015	Pain/discomfort (EQ-5D)	-	169	CD (84), UC (73), Total 157	Total IBD 123	
Piercy et al, 2015	Pain/discomfort (EQ-5D)	2	2065	CD (1084) UC (981)	CD (601) UC (509)	3.32 3.11
Shah et al. 2018.	Pain/discomfort at defecation (SAGIS)	12	10,000	CD (1,157)	CD (141)	0.12
				UC (936)	UC (108)	0.12
Yan et al. 2020.	Pain or discomfort (PRO for IBD)	21	1005	CD (522)	CD (223)	0.24
				UC (363)	UC (176)	0.27
				IBDU (6)		

*Discomfort rates were calculated by taking the number of patients reporting discomfort/sample size and converting it to 12 months.

CD, Crohn’s disease; Dx, diagnosis; Hosp, hospitalization; IBD, inflammatory bowel disease; Mod/sev, moderate to severe disease; UC, ulcerative colitis.

### Determinants

Determinants are a range of reported factors that impact discomfort in adults living with IBD. The determinants of discomfort identified in this systematic review include health literacy (37), disease activity (37,38,41), hospitalization/surgery (37), age and gender (40,41), delayed diagnosis (40), local practice standards (40), and quality of IBD care (45).

#### Health Literacy (HL)

In one included study (37), patients with CD reporting pain/discomfort had a significantly lower median HL (12.5 [10.3–14.8], *P* = 0.02). However, this prospective and observational study did not establish a causal relationship between the two.

#### Disease Activity

The studies included in this systematic review used a range of disease activity indices. A significantly higher median Crohn’s Disease Activity Index (CDAI) has been described in CD patients who faced more difficulties with pain/discomfort (123.6 [89.1–232.3] versus 20.9 [0.0–41.5], *P* < 0.0001) (37). Similarly, more patients (69.9%) with moderate to severe UC experienced pain/discomfort when compared with those in remission (19%), based on a partial Mayo score (PMS) (38). Comparably, an increased odds for moderate to severe vulvar and vaginal discomfort (OR 1.68; 95% CI, 1.22–2.32) has been reported by female participants with active IBD, based on the Manitoba index, when compared with women in remission (41).

#### Hospitalization/Surgery

More patients with CD who required surgery or hospitalization (54%) reported pain/discomfort when compared to those who did not (49%) (37).

#### Age and Gender

Younger female participants’ were more agreeable to respond to inquiries about vulvovaginal symptoms (41). Similarly, more women with CD (805 of 1144 (70%)) completed the vulvar and vaginal symptom survey when compared with women with UC/IC (445 of 690 [65%]), *P* = 0.01. In addition, the participants with perianal discomfort were significantly younger than those reporting other symptoms (40).

#### Delayed Diagnosis

Patients with perianal discomfort had active symptoms for an extended period before their diagnosis was established (40). Perianal discomfort was found to be a significant and only factor associated with a long diagnostic delay (OR 10.23, 95%CI: 1.93–54.37) in Crohn’s disease (*P* = 0.006), but not in UC (40).

#### Local Practice Standards

Patients with CD presenting with perianal discomfort in Korea experienced longer physician-dependent delays the period from initial presentation to diagnosis) (45). The authors attributed these findings to local practice standards, which differ from the West, and include referring patients presenting with perianal discomfort to a surgeon or general practitioner instead of a gastroenterologist as IBD is uncommon in the East (40).

#### Quality of IBD Care

The patient satisfaction with the quality of their IBD care was lower in patients experiencing pain or discomfort in a bivariate analysis evaluating patient-centred outcomes (*P* = 0.043) (45).

## DISCUSSION

This is the first review that systematically synthesized the best available evidence to determine the worldwide incidence, prevalence and determinants of discomfort in adults with IBD. The characteristics of the adult population with IBD experiencing discomfort support the underlying complexities of IBD across disease type, disease activity, age, gender, range of measurement tools, practice standards and relationships with the health care teams. The findings of this review suggest that discomfort is prevalent in adults living with IBD. Therefore, health care professionals providing care to adults with IBD should recognize discomfort as an independent symptom that can impact their quality of life and perception of quality care. Consequently, standardized measurement and evaluation strategies of discomfort should be considered in clinical practice to deliver patient-centred services.

The variability of the studies reporting discomfort in IBD limits our understanding of this symptom. Patient-centred research will allow the exploration of patients’ care priorities and recognize their sources of discomfort or distress (29). Thus, future research efforts should include studies to evaluate the efficacy of discomfort interventions for people with IBD.

## LIMITATIONS

The inclusion of only English-language studies may have led to the exclusion of seminal studies. Given the quality of the included studies, the lack of differentiation between pain and discomfort in the EURO-QOL 5 domain, and the high level of heterogeneity observed during the meta-analysis, the findings of this review must be interpreted with caution. In addition, there was insufficient data to conduct subgroup analyses.

## CONCLUSION

Discomfort is prevalent in adults with IBD. Determinants of discomfort amenable to intervention may include health literacy, disease activity, hospitalization, surgery, age and gender, delayed diagnosis, local practice standards and quality of IBD care. Unfortunately, there is no specific, validated measure of discomfort in IBD. Therefore, a consensus on how discomfort is defined, acknowledged and investigated is recommended to represent patients’ experiences and care needs accurately.

## Supplementary Material

gwab043_suppl_Supplementary_Appendix

## References

[1] Conley S, Proctor DD, Jeon S, et al. Symptom clusters in adults with inflammatory bowel disease. Res Nurs Health 2017;40(5):424–34. 28833284 10.1002/nur.21813 PMC5597486

[2] Dibley L, Norton C, Whitehead E. The experience of stigma in inflammatory bowel disease: An interpretive (hermeneutic) phenomenological study. J Adv Nurs 2018;74(4):838–51. 29105144 10.1111/jan.13492

[3] Ng SC, Shi HY, Hamidi N, et al. Worldwide incidence and prevalence of inflammatory bowel disease in the 21st century: A systematic review of population-based studies. Lancet 2017;390(10114):2769–78. 29050646 10.1016/S0140-6736(17)32448-0

[4] Fourie S, Jackson D, Aveyard H. Living with inflammatory bowel disease: A review of qualitative research studies. Int J Nurs Stud 2018;87:149–56. 30125834 10.1016/j.ijnurstu.2018.07.017

[5] Kaplan GG, Bernstein CN, Coward S, et al. The impact of inflammatory bowel disease in Canada 2018: Epidemiology. J Can Assoc Gastroenterol 2019;2(Suppl 1):S6–S16. 31294381 10.1093/jcag/gwy054 PMC6512243

[6] Coward S, Clement F, Benchimol EI, et al. Past and future burden of inflammatory bowel diseases based on modeling of population-based data. Gastroenterology 2019;156(5):1345–53.e4. 30639677 10.1053/j.gastro.2019.01.002

[7] Damião AOMC, de Azevedo MFC, Carlos AS, et al. Conventional therapy for moderate to severe inflammatory bowel disease: A systematic literature review. World J Gastroenterol 2019;25(9):1142–57. 30863001 10.3748/wjg.v25.i9.1142 PMC6406187

[8] Haig TH, Scott DA, Stevens GB. Measurement of the discomfort component of illness. Med Care 1989;27(3):280–7. 2927184 10.1097/00005650-198903000-00006

[9] Ashkenazy S, DeKeyser Ganz F. The differentiation between pain and discomfort: A concept analysis of discomfort. Pain Manag Nurs 2019;20(6):556–62. 31307870 10.1016/j.pmn.2019.05.003

[10] Mahadev S, Young JM, Selby W, Solomon MJ. Quality of life in perianal Crohnʼs Disease: What do patients consider important? Dis Colon Rectum 2011;54(5):579–85. 21471759 10.1007/DCR.0b013e3182099d9e

[11] Bernhofer EI, Masina VM, Sorrell J, et al. The pain experience of patients hospitalized with inflammatory bowel disease: A phenomenological study. Gastroenterol Nurs 2017;40(3):200–7. 26247627 10.1097/SGA.0000000000000137

[12] Sykes DN, Fletcher PC, Schneider MA. Balancing my disease: Women’s perspectives of living with inflammatory bowel disease. J Clin Nurs 2015;24(15-16):2133–42. 25694255 10.1111/jocn.12785

[13] Vollebregt PF, van Bodegraven AA, Markus-de Kwaadsteniet TML, et al. Impacts of perianal disease and faecal incontinence on quality of life and employment in 1092 patients with inflammatory bowel disease. Aliment Pharmacol Ther 2018;47(9):1253–60. 29520808 10.1111/apt.14599 PMC5947114

[14] Sexton KA, Walker JR, Targownik LE, et al. The inflammatory bowel disease symptom inventory: A patient-report scale for research and clinical application. Inflamm Bowel Dis 2019;25(8):1277–90. 30918969 10.1093/ibd/izz038 PMC6635838

[15] Sammut J, Scerri J, Xuereb RB. The lived experience of adults with ulcerative colitis. J Clin Nurs 2015;24(17-18):2659–67. 26111098 10.1111/jocn.12892

[16] Falt P, Šmajstrla V, Fojtík P, et al. Carbon dioxide insufflation during colonoscopy in inflammatory bowel disease patients: A double-blind, randomized, single-center trial. Eur J Gastroenterol Hepatol 2017;29(3):355–9. 27845950 10.1097/MEG.0000000000000791

[17] Mosli MH, Bukhari LM, Khoja AA, et al. Inflammatory bowel disease and restless leg syndrome. Neurosciences 2020;25(4):301–7. 33130811 10.17712/nsj.2020.4.20200021 PMC8015615

[18] Lim HS, Kim SK, Hong SJ. Food elimination diet and nutritional deficiency in patients with inflammatory bowel disease. Clin Nutr Res 2018;7(1):48–55. 29423389 10.7762/cnr.2018.7.1.48 PMC5796923

[19] Holst M, Jensen HN, Bach U, Fallingborg J, Rasmussen HH. Dietary habits in patients with ulcerative colitis cause of nutrient deficiency? Clin Nutr Suppl 2012;7(1):234–5.

[20] Norton C, Czuber-Dochan W, Artom M, et al. Systematic review: Interventions for abdominal pain management in inflammatory bowel disease. Aliment Pharmacol Ther 2017;46(2):115–25. 28470846 10.1111/apt.14108

[21] Monica F, Canaletti C, Paolini A, Pecoraro G, Paulon E, Tonello C. Psycho-social characteristics and distress risk in the IBD population: A pilot study. Dig Liver Dis 2018;50(2 Supplement 1):e195–e6.

[22] Lönnfors S, Vermeire S, Greco M, Hommes D, Bell C, Avedano L. IBD and health-related quality of life — discovering the true impact. J Crohns Colitis 2014;8(10):1281–6. 24662394 10.1016/j.crohns.2014.03.005

[23] Palant A, Himmel W. Are there also negative effects of social support? A qualitative study of patients with inflammatory bowel disease. BMJ Open 2019;9(1):e022642. 10.1136/bmjopen-2018-022642 PMC6347904 30670504

[24] Saunders B . Stigma, deviance and morality in young adults’ accounts of inflammatory bowel disease. Sociol Health Illn 2014;36(7):1020–36. 24888541 10.1111/1467-9566.12148

[25] Ona S, James K, Ananthakrishnan AN, et al. Association between vulvovaginal discomfort and activity of inflammatory bowel diseases. Clin Gastroenterol Hepatol 2020;18(3):604–11.e1. 31108226 10.1016/j.cgh.2019.05.018

[26] Barros J, Baima J, Renosto F, et al. Inflammatory bowel disease affects sexual female desire and sexual female excitement. J Crohns Colitis 2017;11(Supplement 1):S491.

[27] Kara B, Ozenli Y, Yalaki S. Exaggeration of physical symptoms in inflammatory bowel disease: Relation to depression, anxiety and quality of life. J Crohns Colitis 2014;8(Suppl. 1):S331.

[28] Ruan J, Chen Y, Zhou Y. Development and validation of a questionnaire to assess the quality of life in patients with inflammatory bowel disease in Mainland China. Inflamm Bowel Dis 2017;23(3):431–9. 28129287 10.1097/MIB.0000000000001024

[29] Armuzzi A, Riegler G, Vecchi M, et al. Epidemiological features and disease related concerns of a large cohort of Italian patients with active Crohn’s disease. J Crohns Colitis 2019;8(Suppl. 1):S332. 10.1016/j.dld.2018.12.019 30685416

[30] Munn Z, Moola S, Lisy K, et al. Methodological guidance for systematic reviews of observational epidemiological studies reporting prevalence and cumulative incidence data. Int J Evid Based Healthc 2015;13(3):147–53. 26317388 10.1097/XEB.0000000000000054

[31] Page MJ, McKenzie JE, Bossuyt PM, et al. The PRISMA 2020 statement: An updated guideline for reporting systematic reviews. BMJ 2021;372:n71. 33782057 10.1136/bmj.n71 PMC8005924

[32] Nistor OI, Rosemary W, Godfrey C, Ross-White A. Incidence, prevalence, and determinants of discomfort in inflammatory bowel disease: A systematic review and meta-analysis protocol. In: PROSPERO, 2020. https://www.crd.york.ac.uk/prospero/display_record.php?RecordID=226061 10.1093/jcag/gwab043 PMC8972280 35368319

[33] Haddaway NR, Collins AM, Coughlin D, et al. The role of Google Scholar in evidence reviews and its applicability to Grey literature searching. PLoS One 2015;10(9):e0138237. 26379270 10.1371/journal.pone.0138237 PMC4574933

[34] Aromataris E, Munn Z, (eds). JBI Manual for Evidence Synthesis. JBI, 2020. https://jbi-global-wiki.refined.site/space/MANUAL

[35] Munn Z, Aromataris E, Tufanaru C, et al. The development of software to support multiple systematic review types: The Joanna Briggs Institute System for the Unified Management, Assessment and Review of Information (JBI SUMARI). Int J Evid Based Healthc 2019;17(1):36–43. 30239357 10.1097/XEB.0000000000000152

[36] Moola S, Munn Z, Tufanaru C, et al. Chapter 7: Systematic reviews of etiology and risk. In: JBI Manual for Evidence Synthesis. JBI, 2020. https://jbi-global-wiki.refined.site/space/MANUAL/3283910762/Chapter+7%3A+Systematic+reviews+of+etiology+and+risk

[37] Carels C, Wauters L, Baert F, et al. Health literacy, quality of life, work productivity and activity impairment in young adults with Crohn’s disease compared to diabetes mellitus patients: Long-term follow-up from the Belgian Crohn’s disease registry. United Eur Gastroenterol J 2019;7(8 Supplement):960.

[38] Ding Z, Obando C, Izanec J, et al. Relationship between partial mayo score and patient-reported outcomes for ulcerative colitis patients in the United States. Am J Gastroenterol 2019;114:S417–S8.

[39] Obando C, Ding Z, Izanec J, et al. Relationship between Crohn’s disease activity index and patient-reported outcomes for Crohn’s disease patients in the United States. Am J Gastroenterol 2019;114:S445.

[40] Lee DW, Koo JS, Choe JW, et al. Diagnostic delay in inflammatory bowel disease increases the risk of intestinal surgery. World J Gastroenterol 2017;23(35):6474–81. 29085197 10.3748/wjg.v23.i35.6474 PMC5643273

[41] Ona S, James K, Ananthakrishnan AN, et al. Association between vulvovaginal discomfort and activity of inflammatory bowel diseases. Clin Gastroenterol Hepatol 2020;18(3):604–11.e1. 31108226 10.1016/j.cgh.2019.05.018

[42] Petryszyn P, Zachariasz A, Ekk-Cierniakowski P, Well M. Health-related quality of life in patients with inflammatory bowel disease in Poland (application of the EQ-5D and self-assessment of health state). Value Health 2015;18(7):A629.

[43] Piercy J, Ozbay AB, Roughley A, Chao J, Skup M. Comparison of ulcerative colitis with Crohn’s disease from the perspective of patient burden. Gastroenterology 2015;148(4 Suppl. 1):S839.

[44] Shah A, Rich J, Ghasemi P, et al. Gastrointestinal symptoms impacting on quality of life: A comparative cohort study in patients with organic and functional gastrointestinal disorders (FGIDS) in the Tertiary Hospital outpatient setting. United Eur Gastroenterol J 2018;6(8 Supplement):A320–A1.

[45] Yan X, Qiao Y, Tong J, et al.; Young Investigators for the study of Inflammatory Bowel Diseases (YIIBD). Assessment of patient-centered outcomes (PROs) in inflammatory bowel disease (IBD): A multicenter survey preceding a cross-disciplinary (functional) consensus. Health Qual Life Outcomes 2020;18(1):241. 32690091 10.1186/s12955-020-01489-8 PMC7372780

[46] Munn Z, Moola S, Lisy KRD, Tufanaru C. Chapter 5: Systematic Reviews of Prevalence and Incidence. JBI, 2020. https://jbi-global-wiki.refined.site/space/MANUAL/3283910689/Chapter+5%3A+Systematic+reviews+of+prevalence+and+incidence

