# Supplementary material for: Systematic Review and Meta-analysis on the Incidence, Prevalence and Determinants of Discomfort in Inflammatory Bowel Disease
Source: J Can Assoc Gastroenterol. 2021 Nov 14;5(2):96–7. doi: 10.1093/jcag/gwab043 (PMC8972280; doi:10.1093/jcag/gwab043)
Supplement: gwab043_suppl_Supplementary_Appendix [file gwab043_suppl_supplementary_appendix.docx]

**Appendix I: Search strategy Medline, Embase and PsychInfo**

Database: Ovid MEDLINE(R), Ovid MEDLINE(R) Daily and Epub Ahead of Print, In-Process & Other Non-Indexed Citations <1946 to Present>

Search Strategy:

--------------------------------------------------------------------------------

1 exp Inflammatory Bowel Diseases/ (82475)

2 inflammatory bowel disease*.ti,ab,kw. (51633)

3 crohn*.ti,ab,kw. (49451)

4 colitis.ti,ab,kw. (67048)

5 ibd.ti,ab. (26071)

6 or/1-5 (136737)

7 discomfort*.mp. (48068)

8 6 and 7 (389)

9 limit 8 to english language (352)

10 limit 9 to animals (39)

11 9 not 10 (313)

Database: Embase Classic+Embase <1947 to 2021 January 08>

Search Strategy:

--------------------------------------------------------------------------------

1 exp Inflammatory Bowel Diseases/ (165536)

2 inflammatory bowel disease*.ti,ab,kw. (87599)

3 crohn*.ti,ab,kw. (87189)

4 colitis.ti,ab,kw. (116317)

5 ibd.ti,ab. (55175)

6 or/1-5 (233170)

7 discomfort*.mp. (89542)

8 6 and 7 (1420)

9 limit 8 to english language (1351)

10 limit 9 to animals (33)

11 9 not 10 (1318)

Database: APA PsycInfo <1806 to January Week 1 2021>

Search Strategy:

--------------------------------------------------------------------------------

1 ibd.ti,ab. (558)

2 discomfort*.mp. (10804)

3 irritable bowel syndrome/ or exp colitis/ (1618)

4 inflammatory bowel disease*.ti,ab. (907)

5 crohn*.ti,ab. (607)

6 colitis.ti,ab. (941)

7 1 or 3 or 4 or 5 or 6 (2941)

8 2 and 7 (76)

9 limit 8 to english language (74)

**Appendix II: Search strategy CINAHL**
